# The DERIVO 2 Heal Embolization Device in the Treatment of Ruptured and Unruptured Intracranial Aneurysms: a Retrospective Multicenter Study

**DOI:** 10.1007/s00062-024-01446-8

**Published:** 2024-08-22

**Authors:** Roland Schwab, Christoph Kabbasch, Lukas Goertz, Marius Kaschner, Daniel Weiss, Christian Loehr, Hauke Wensing, Maxim Bester, Andreas Simgen, André Kemmling, Christina Wendl, Erelle Fuchs, Maximilian Thormann, Daniel Behme, Hannes Nordmeyer

**Affiliations:** 1 University Clinic for Neuroradiology, Medical Faculty and University Hospital, Otto-von-Guericke-University Magdeburg, Magdeburg, Germany; 2 Department of Radiology and Neuroradiology, Faculty of Medicine and University Hospital, University of Cologne, Cologne, Germany; 3 Department of Diagnostic and Interventional Radiology, Medical Faculty, University Düsseldorf, Düsseldorf, Germany; 4 Department of Radiology and Neuroradiology, Klinikum Vest, Recklinghausen, Germany; 5 Department of Diagnostic and Interventional Neuroradiology, University Medical Center Hamburg-Eppendorf, Hamburg, Germany; 6 Department for Neuroradiology, Westpfalz-Klinikum Kaiserslautern, Kaiserslautern, Germany; 7 Department of Neuroradiology, University Marburg, Marburg, Germany; 8 University Hospital Regensburg, Institute of Radiology, Regensburg, Germany; 9 Department of Neuroradiology Städtisches Klinikum Solingen, Solingen, Germany; 10 Research Campus STIMULATE, Otto-von-Guericke University Magdeburg, Magdeburg, Germany; 11 Medical School, Department of Health, Witten/Herdecke University, Witten, Germany

**Keywords:** SAH, hemorrhage, flow diverter, endovascular treatment, cerebral aneurysm

## Abstract

**Backround:**

The use of flow diverting stents in the treatment of intracranial aneurysms is associated with a risk of neurological morbidity due to their thrombogenicity. To reduce this risk different surface modifications have been developed. The Derivo 2 Embolization Device (Acandis, Pforzheim, Germany) has proven to be a safe and effective flow diverter. To overcome the risk of thrombo-embolism, the device was modified by adding an anti-thrombogenic fibrin-heparin coating. We aimed to assess the safety and effectiveness of the Derivo 2 heal Embolization Device.

**Methods:**

Retrospective multicenter data from nine German neurovascular centers between February 2022 until December 2023 were used. Patients treated with the Derivo 2 heal Embolization Device for unruptured or ruptured intracranial aneurysms were included. Peri- and postprocedural adverse events, clinical outcomes, and angiographic follow-up results were evaluated.

**Results:**

84 patients (73.8% female; mean age 58.7 years) with 89 aneurysms (mean size 9.8 mm) were included. 87.6% were located in the anterior circulation. Most of them were sidewall aneurysms (88.8%). 96 flow diverters were used. 99.0% were successfully implanted. An in-stent balloon angioplasty was performed in 6.0% of the cases. An additional coiling was performed in 28.6%. Technical difficulties were present in 12.0% of the cases. Thrombotic events occurred in 4.8% with no neurological sequelae. Mortality and morbidity were 0 and 1.2% respectively. Adequate aneurysm occlusion was achieved in 80.7% with a mean follow-up time of 6.6 months.

**Conclusion:**

The Derivo 2 heal Embolization Device showed a satisfying aneurysm occlusion and safety with a low rate of neurological morbidity.

**Supplementary Information:**

The online version of this article (10.1007/s00062-024-01446-8) contains supplementary material, which is available to authorized users.

## Introduction

The use of flow diversion has emerged over the last decade. Initially, the main use was for the treatment of wide-neck aneurysms of large cerebral vessels. Nowadays, different types of aneurysms like dissecting, fusiform, and blister-like aneurysms, or even small cerebral vessel aneurysms can be treated via flow diversion with an acceptable rate of procedure-related morbidity and favorable outcomes [1–3]. High thrombogenicity due to low porosity and high mesh density of flow diverting stents (FD) is described as one of the main reasons for neurological morbidity after flow diversion and requires a sufficient dual antiplatelet therapy (DAPT) for several months post procedure [1, 2, 4]. This circumstance limits the usage of FD in acutely ruptured aneurysms due to a higher risk of hemorrhagic complications or in case of possible interference of necessary medication like oral anticoagulation [5, 6]. Manufacturers developed different surface modifications like Shield Technology (Medtronic, Minneapolis, Minnesota, USA), HPC (Phenox, Bochum, Germany), and X Technology (Microvention, Aliso Viejo, California, USA), to reduce thrombogenicity [7].

The HEAL Technology (Acandis, Pforzheim, Germany) mimics the final step of natural hemostasis and thus promotes the conversion of fibrinogen to fibrin. The controlled growth process results in a thin and fully polymerized network of fibrin with additional covalently bound heparin molecules. This composition of the HEAL Coating constitutes the antithrombogenic and endothelialization promoting properties that are essential for a rapid healing process of an intracranial device. Fibrin passivates the surface so that the implant cannot be perceived as a foreign body and neither inflammatory nor coagulative processes are triggered. Passivation thus leads to a significant reduction in thrombogenicity [8]. At the same time, the fibrin network forms a scaffold for surrounding endothelial cells and may support endothelialization and healing of the implant in the vessel. The covalently bound heparin reduces platelet activation and activation of the coagulation cascade. Thus, heparin also contributes to the antithrombogenic property of the HEAL Coating. Neither heparin nor any other components of the coating are released after implantation of the device. Thus, the coating is not elusive and has no systemic pharmacological effect [9]. Previous studies have shown high occlusion rates with low adverse event rates for the Derivo and the Derivo 2 Embolization Device [10, 11]. Our study evaluates early clinical and angiographic outcomes of ruptured and unruptured aneurysms treated with the Derivo 2 heal Embolization Device (D2H; Acandis GmbH, Pforzheim, Germany).

## Materials and Methods

### Study Design

Multicentric retrospective data from nine neurovascular centers in Germany were used for this study. All patients with ruptured or unruptured intracranial aneurysm, treated with the new D2H in the limited market use from February 2022 until October 2023 were included. The patient clinical data, demographics, aneurysm characteristics, procedural details and follow-up data were analyzed retrospectively from prospectively collected data. The clinical patient data and postprocedural measurements were observed from February 2022 until December 2023. Ethical approval was obtained as required according to the guidelines of the local ethics committees (Ethics Committee, Medical Faculty of Otto-von-Guericke-University Magdeburg, Magdeburg, Germany). This anonymous retrospective study, which was conducted in accordance with the Declaration of Helsinki, fulfils the guidelines of the federal state of Saxony-Anhalt. The data has been legally collected in accordance with § 15 paragraph [5] (University Hospital Magdeburg).

### Clinical Data and Outcome

Presentation was defined as asymptomatic, acute subarachnoid hemorrhage (aSAH), compressive syndromes or other (headache, pulsatile tinnitus, vertigo). The modified Rankin scale (mRS) was used to measure pre- and postprocedural patient outcome (0–2 favorable outcome; 3–5 major morbidity) [12]. In case of ruptured intracranial aneurysms, the prerupture mRS was additionally assessed. The National Institutes of Health Stroke Scale score (NIHSS) was evaluated preprocedural and in the case of neurological deterioration [13].

### Aneurysm Data

Topography, location (sidewall, bifurcation), aneurysm type (saccular, blister like, fusiform, dissecting, mycotic), arising branches of the aneurysm, maximum diameter (mm), neck width (mm) and neck-dome ratio was assessed.

### Procedure

Indication for treatment was determined and performed by a board certified neurointerventionalist in consensus with the local neurovascular board. The primary use of the D2H (Acandis GmbH, Pforzheim, Germany) without size restriction was mandatory. All procedures were performed under general anesthesia. Periprocedural heparinization was left to the discretion of the treating physician. Additional use of coils, a necessity of post-implantation balloon angioplasty, as well as use of other devices were assessed.

Successful device delivery over the microcatheter and technical difficulties like incomplete device opening, fish mouthing phenomenon, device displacement and device twisting were assessed. Clinical examination was performed 24 h before and after treatment, at discharge, and during follow up visits. Clinical deterioration that resolved in between 7 days or events without clinical sequelae were classified as minor adverse events. Deficits lasting longer than 7 days were defined as major adverse events. Symptomatic intracranial hemorrhage (sICH) was defined as a new parenchymal or subarachnoid hemorrhage with neurological deterioration postprocedural up until day 14. Major ischemic events were defined as an increase of 4 or more NIHSS points for > 24 h.

### Antiplatelet Therapy

Testing of antiplatelet therapy response and pre-, peri- and postprocedural antiplatelet aggregation therapy was performed according to local institutional standards. The type of medication and the duration were assessed.

### Degree of Aneurysm Occlusion

The aneurysm occlusion was angiographically rated using the O’Kelly-Marrota Scale (OKM; A = total filling; B = subtotal filling; C = entry remnant; D = no filling). Adequate aneurysm occlusion was defined as OKM C‑D [14].

### Follow-up

Follow-up (FU) was performed according to local standards. For the rating of the degree of aneurysm occlusion only digital subtraction angiography (DSA) imaging was accepted and assessed by the treating institution. In addition to the mRS, the appearance of an In-Stent stenosis (ISS), In-Stent thrombosis, aneurysm rupture, and performed retreatment were assessed. The degree of ISS was measured in comparison to the postprocedural DSA imaging. A degree of 0% was described as “not present”, ≤ 50% “mild”, 50–75% “moderate” and > 75% severe.

### Statistical Analysis

For the statistical analysis SPSS 29 (IBM, Redmond) was used. The clinical and patient data, aneurysm characteristics and outcome parameter were analyzed descriptively. Continuous variables are listed as mean, ± standard deviation (*SD*) and range. Binary variables are listed as total numbers and percentage.

Differences between two groups of binary variables were calculated with the χ2 test.

Depending on the normal distribution, the unpaired t‑test or the Mann-Whitney U‑test was used for differences between two groups for continuous variables. Statistical significance was described as *p*-values < 0.05.

## Results

84 Patients with 89 intracranial aneurysms were included. At presentation, 81 aneurysms were unruptured, and 8 were ruptured. The results, subdivided into unruptured and ruptured aneurysms, are summarized in the online supplemental data.

### Patient and Aneurysm Characteristics

The mean age was 58.7 years (*SD* ± 14.39; 17–90). 73.8% were female. 87.6% of the aneurysms were located in the anterior circulation, with the majority located in the internal carotid artery (83.1%). 68.6% were sidewall aneurysms. In 62.9% the morphology was saccular, 20.2% fusiform, 9.0% dissecting and 7.9% blister like. The mean size was 9.8 mm (*SD* ± 6.9 [1.8–45]) with a mean neck width of 5.5 mm (*SD* ± 3.9 [1.5–25]) and a mean dome-to-neck ratio of 1.6 (*SD* ± 0.9 [0.1–8]). The presentation was in 63.1% (*n* = 53) asymptomatic, 8 patients (9.5%) had an aSAH, 7 patients (8.3%) had compressive syndromes, 4 patients (4.8%) had a recurrent aneurysm after coiling, stent assisted coiling or failed clipping, and 12 patients (14.3%) had other symptoms (headache, gait, TIA, stroke). Patient and aneurysm characteristics are summarized in Table 1.

**Table 1:** Baseline patient and aneurysm characteristics

Characteristics	Values
Age (years) (mean ± *SD* [range])	58.7 ± 14.39 (17–90)
*Sex (n [%])*	
Female	62 (73.8)
Male	22 (26.2)
*Baseline mRS (n [%])*	
0–2	83 (98.8)
3–5	1 (1.2)
*Medical History (n [%])*	
Arrhythmia/Atrial fibrillation	81 (96.4)
Diabetes Mellitus	77 (91.7)
Dyslipidemia	55 (65.5)
Hypertension	40 (47.6)
Obesity	72 (58.7)
Smoking	65 (77.4)
*Presentation (n [%])*	
Asymptomatic	53 (63.1)
aSAH	8 (9.5)
Recurrent aneurysm	4 (4.8)
Compressive Symptoms	7 (8.3)
Other	12 (14.3)
*Size (mm) (mean* *±* *SD [range])*	*9.76* *±* *6.97 (1.8–45)*
< 5 mm (*n* [%])	18 (20.2)
> 5 < 10 mm (*n* [%])	35 (39.3)
> 10 < 25 mm (*n* [%])	33 (37.1)
> 25 mm (*n* [%])	3 (3.4)
Neck width (mm) (mean ± *SD* [range])	5.47 ± 3.96 (1.5–25)
Dome-Neck ratio (mean ± *SD* [range])	1.57 ± 0.98 (0.1–8)
*Site (n [%])*	
Right	45 (50.6)
Left	39 (43.8)
Midline	5 (5.6)
*Location (n [%])*	
Bifurcation	10 (11.2)
Sidewall	61 (68.6)
Segmental	18 (20.2)
*Morphology (n [%])*	
Blister like	7 (7.9)
Dissecting	8 (9.0)
Fusiform	18 (20.2)
Saccular	56 (62.9)
***Topography (n [%])***	
**Anterior circulation**	
*ICA*	*74 (83.1)*
Carotid‑T	2 (2.2)
Cavernous	10 (11.2)
Anterior choroidal	1 (1.1)
PCOM origin	15 (16.8)
Paraopthalmic	42 (47.2)
Petrous	4 (4.5)
*MCA bifurcation*	*3 (3.3)*
*ACA*	*1 (1.1)*
**Posterior circulation**	
Basilar	5 (5.6)
PICA	2 (2.2)
SCA	1 (1.1)
VA (V4)	3 (3.3)

*mRS* modified Rankin scale, *aSAH* acute subarachnoid hemorrhage, *ICA* internal carotid artery, *PCOM* posterior communicating artery, *MCA* middle cerebral artery, *ACA* anterior cerebral artery, *PICA* posterior inferior cerebeallar artery, *SCA* superior cerebellar artery, *VA* vertebral artery

### Antiplatelet Therapy

A DAPT was carried out in 98.8% of cases (*n* = 83). Only one Patient had a single antiplatelet therapy (SAPT) with Clopidogrel due to a former allergic reaction to acetylsalicylic acid (ASA). The most common combination was ASA and Clopidogrel (67.9%; *n* = 57) followed by ASA and Prasugrel (16.7%; *n* = 14). The mean duration of the DAPT was 6.3 month (*SD* ± 3.3 [3–20]). Detailed overview is provided in the online supplemental data. After DAPT, 21.4% (*n* = 18) of patients switched to permanent SAPT, 78.6% did not receive any further antiplatelet therapy. The most commonly administered medication was ASA (88.9%), followed by Clopidogrel (11.1%).

### Treatment

Overall, 96 FD were used. 1 FD was used in 76 cases (90.4%), 2 FD in 5 cases (6.0%), 3 FD in 2 cases (2.4%) and 4 FD in 1 case (1.2%). An additional coiling was performed in 24 cases (28.6%). The mean aneurysm size was significantly higher in the additional coiling group (11.2 mm *SD* ± 4.7 [5.7–24] vs 9.2 mm *SD* ± 7.6 [1.8–45]; [*p* = 0.04]). Furthermore, the aneurysm morphology differed significantly (*p* = 0.03). The most common morphology observed in the additional coiling group was saccular aneurysms, accounting for 79.2% of cases, compared to 56.9% in the non-additional coiling group. Fusiform aneurysms were the second most frequent morphology, with a higher prevalence in the non-additional coiling group (21.5% vs. 16.7%). There was no significant difference between both groups in neck width, age and gender (*p* = 0.41; *p* = 0.72; *p* = 0.52). An in-stent balloon angioplasty was performed in 5 cases (6.0%) in order to optimize the wall apposition. In 99.0% (*n* = 95) of the cases, the device could be delivered successfully over the microcatheter. In one case, the D2H (6 × 40 mm) could not be delivered due to high friction in the microcatheter (Headway 27, Microvention, Aliso Viejo, California, USA) as a result of tortuous anatomy. A resheathing and replacement of the FD was necessary in 18 cases (21.4%). In 3 cases (3.6%) the FD twisted. A fish mouthing occurred in 4 cases (4.8%). In 2 Cases (2.4%) the FD did not fully open (< 50%). In those cases, the FD proximally did not open and a high-grade fish mouthing phenomenon immediately became apparent. An additional overlapping stent or flow diverter implantation (Credo heal Stent, 4.0 × 25 mm; Acandis, Pforzheim, Germany; Derivo Flow Diverter, 5 × 25 mm, Acandis, Pforzheim, Germany) was performed and completely solved the difficulty. None of the mentioned technical difficulties had clinical sequelae. The postprocedural aneurysm occlusion grade was OKM A in 59.5% (*n* = 50), OKM B in 23.8% (*n* = 20), OKM C in 6.0% (*n* = 5) and OKM D in 10.7% (*n* = 9) of the cases. There was no postprocedural mRS shift compared to the baseline mRS.

### Complications

An intraprocedural thrombotic event occurred in 4 cases (4.8%). In 2 cases they were located inside the FD and in 2 cases a distal thromboembolism appeared. All thrombotic events were completely resolved under administration of local intraarterial Glykoprotein-IIb/IIIa-Receptor-Antagonists (Tirofiban or Eptifibatid). In 7.1% (*n* = 6) of the cases punctate embolic infarcts showed up in the postprocedural MRI scan with no clinical sequelae. There were no major ischemic events. 3 patients (3.6%) suffered a sICH. During the procedure, an unruptured aneurysm ruptured due to additional coiling. There was 1 (1.2%) asymptomatic retroperitoneal hematoma and 1 (1.2%) groin hematoma located at the femoral puncture site. 1 patient suffered a contrast induced encephalopathy (CIE). The procedure was performed with 100 ml of Ultravist 370 (Bayer Vital, Leverkusen, Germany). Symptoms improved after 24 h. No medical treatment was given. The neurological symptoms resolved completely by discharge. Technical difficulties and adverse events are summarized in Table 2.

**Table 2:** Technical difficulties and adverse events

Characteristics	Values (*N* = 84)
*Technical difficulties (n [%])*	*10 (12)*
FD did not open	2 (2.4)
FD not deliverable	1 (1.2)
FD twisted	3 (3.6)
Fish mouthing	4 (4.8)
*Minor adverse events (n [%])*	*7 (8.4)*
Intraproc. thrombotic event	4 (4.8)
Hematoma at puncture site	2 (2.4)
CIE	1 (1.2)
*Major adverse events (n [%])*	*4 (4.8)*
sICH	3 (3.6)
Intraproc. aneurysm rupture	1 (1.2)
Major ischemic event	0 (0)
*Mortality (n [%])*	*0 (0)*
*Morbidity(n [%])*	*1 (1.2)*

*FD* flow diverter, *CIE* contrast induced encephalopathy, *sICH* symptomatic intracranial hemorrhage

### Follow-up

**Fig. 1:**
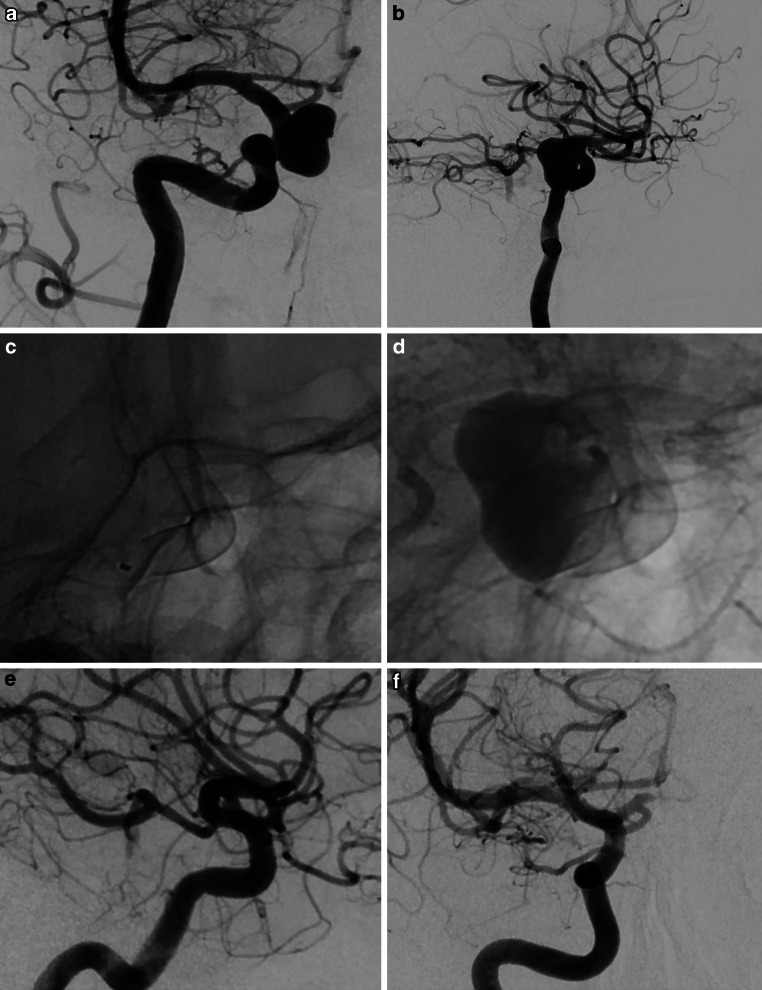
Patient in their 50s with an incidental, right sided, saccular, paraopthalmic ICA sidewall aneurysm (max. size 14 mm; neck width 4 mm). Pretreatment angiogram in anteroposterior (**a**) and lateral (**b**) projection. **c** Single shot after deployment of a 4 × 15 mm Derivo 2 heal flow diverter (Acandis, Pforzheim, Germany). **d** Instant unsubstracted angiogram in lateral projection with already intraaneurysmal stasis. **e**, **f** Full aneurysm occlusion after 6 months with smooth remodeling of the parent vessel

78 patients (92.9%) had a FU including at least one DSA FU imaging. At the time of follow-up, six patients had dropped out. The mean time to FU was 6.6 months (*SD* ± *3.59* [1–18]). A retreatment was performed in 2 cases (2.4%). There was no aneurysm re-rupture during the FU period. 80.7% (*n* = 63) of the FU cases had an adequate aneurysm occlusion (OKM C‑D). An illustrative case is shown in Fig. 1. There was no In-Stent thrombosis. Overall, 12 Patients (15.4%) had an ISS of varying degrees. None of the ISS were treated. In 5 cases the ISS completely resolved during the FU period back down to a stenosis degree of 0%. 7 patients (8.9%) showed an ISS during the last FU DSA imaging. 6 had a mild ISS (< 50%) and 1 patient had a moderate ISS (55%). The course of the ISS is shown in online supplemental data. Compared to the subgroup with no additional coiling (*n* = 60), the rate off ISS was significantly higher (*p* = 0.02) in the subgroup with an additional coiling (*n* = 24; visualized in online supplemental data). One patient had a mRS shift overall compared to the baseline mRS. This patient had an unruptured aneurysm and an initial mRS of 0. He suffered a CIE postprocedural. In the postprocedural MRI small embolic infarcts were revealed. There was no bleeding. The symptoms resolved completely by the discharge date. The DAPT medication consisted of ASA and Clopidogrel. After 14 days he had a mild trauma and presented with a sICH located distantly from the aneurysm and infarctions. The final mRS score was 5. Overview of the FU data in Table 3.

**Table 3:** Follow-up charachteristics

Characteristics	Values (*N* = 78)
Time to FU (month) (mean ± *SD* [range])	6.6 (± 3.59 [1–18])
*OKM (n [%])*	
A -B	15 (19.3)
C‑D	63 (80.7)
*mRS (n [%])*	
0–2	77 (98.7)
3–5	1 (1.3)
Rupture (*n* [%])	0
Retreatment (*n* [%])	2 (2.6)
In-stent thrombosis (*n* [%])	0
*In-stent stenosis on last FU (n [%])*	
Mild	6 (7.7)
Moderate	1 (1.3)
Severe	0

*FU* follow-up, *OKM* O’Kelly-Marrota Scale, *mRS* modified Rankin scale

## Discussion

To our knowledge, we present the largest multicentric study post flow diversion with the Derivo Embolization Device 2 including HEAL surface modification.

The main goal of surface modification is to reduce thrombogenicity in order to lowering rates of major ischemic events and neurological morbidity. For the uncoated 1st Generation predecessor Derivo Embolization Device (Acandis, Pforzheim, Germany) the frequency of major strokes was observed in the prospective multicenter study of Taschner et al. (119 patients) and Trivelato et al. (146 patients) of 4.2 and 1.4%. In respect to our smaller cohort (84 patients), the heterogeneity of the population and different study design, our evaluated frequency of 0% major strokes is lower [10, 11]. Also the overall morbidity with a frequency of 1.2% is lower compared to the former observed data with a frequency of 2.4–12% [10, 11, 15–17]. The favorable aneurysm occlusion rate in this study was 80.7%, slightly lower than the results reported in the previous study of the direct precursor without HEAL coating (Derivo 2 Embolization Device; Acandis, Pforzheim, Germany) by Thormann et al. (85.2%) in 37 patients and the multicentric Derivo Embolization Device data of Chagas Lourenço et al. (87.3%) in 127 patients at the 6‑month follow-up. Both studies had lower proportions of larger aneurysms (> 10 mm) at 29.7 and 23.2%, respectively, compared to 40.5% in our cohort [17, 18]. This difference should explain the mild lower occlusion rates, because larger aneurysms treated with flow diversion tend to have lower occlusion rates in early FU and improve over time [19, 20]. Our data show an ISS in 12 patients (15.4%) going down to 7 patients (8.9%) during the last DSA FU. The majority had a mild ISS (< 50%). Only one patient (1.3%) had a moderate stenosis (55%). Most of the ISS occurred during the early phase in the first 5 months after procedure and they improved or disappeared spontaneously during the first 6–12 months. This is a commonly known phenomenon [21, 22]. Our data is in line with the data of the Diversion-p64 study. Bonafe et al. observed in overall 420 patients treated with the p64 flow modulation device (Phenox, Bochum, Germany) an ISS of varying degrees in 15.4% during the FU between 3–6 months post treatment. The frequency of ISS dropped during the FU at 7–12 months to 8.7%. Except for one case, all residual ISS were mild (< 50%) [21].

An interesting finding is the significantly higher ISS rate (*p* = 0.02) in the subgroup where additional coiling (*n* = 24) was performed compared to the non-additional coiling group (*n* = 60). Due to the small sample size and possible selection bias, the finding may be random. To our knowledge there is no data providing comparable results and an explanation of a possible pathomechanism causing this finding. Perhaps the removal of the jailed microcatheter causes intimal injury, which, combined with the additional intimal damage caused by the placed flow diverter, leads to an overwhelming healing reaction and intimal hyperplasia [23–25]. Another explanation depends on one of the theories of endothelialization after flow diversion treatment. The resulting lower velocity, shear stress, and inflow rate induces an inflammatory milieu that leads to a prothrombotic, activated vascular endothelium and induces parent vessel remodeling [26]. Perhaps this process is accelerated by the faster intraaneurysmal thrombosis due to the additional coiling, resulting in an overreaction and temporary initimal hyperplasia [27]. Compared to its coated competitors, the D2H ranks among the current devices with comparable results. Vollherbst et al. presented the data of the FRESH study in 161 patients for the treatment of IA with the FRED X (Microvention, Aliso Viejo, California, USA) with an antithrombotic surface modification. The adequate aneurysm occlusion during the last FU was 83.1%, an additional coiling was performed in 29.8% of the cases, and minor adverse events occurred in 13% of the cases. Those results are in line with our data. The mortality (1.2%) and neurological morbidity (1.9%) is slightly higher than in our cohort [28]. De Villiers et al. recently published their data of the Vanguard study, using the Pipeline Vantage flow diverter with Shield Technology (Medtronic, Minneapolis, Minnesota, USA) in 101 consecutive cases. In most cases the treated aneurysms had a size < 10 mm which differs to our data (83.4% vs 59.5%). The 6 month occlusion rate was 81.7%, with an overall neurological adverse event rate of 7% and a morbidity of 4.9%. In 6 cases (5.9%), an ISS higher 50% was still present during the last FU [29]. Ernst et al. investigated the multicentric data of IA treatment in 100 patients with the p64MW hydrophilic polymer coated flow modulation device (p64MW-HPC; Phenox, Bochum, Germany) [30]. With a frequency of 84%, the amount of small aneurysms, < 10 mm, was higher than in our cohort (59.5%). The adequate occlusion is with 93% at a mean FU time of 7 months (*SD* ± 3) higher compared to our observed data. We believe this depends primarily on the higher proportion of smaller aneurysms, as previously mentioned. Technical adverse events occurred slightly more often in 14% of the cases. They observed a higher rate of severe thrombotic events which caused a neurological deficit that led to morbidity (4% vs 0%). At 19%, ISS occurred more frequently compared to our study.

In general, the current coated FD generation of different manufacturers show comparable results to their uncoated predecessors. In some points, the differences are nuanced. A real superiority is actually not shown [18, 28–33]. In our opinion, a deeper look is required to reveal more in-depth differences. The possibility of a SAPT would be beneficial and would expand the use of flow diversion. According to the updated IFU, the use of D2H under SAPT with Prasugrel is possible in selected cases. In general, there is not yet sufficient evidence for a possible SAPT using HEAL coated devices. Nowadays only retrospective data or case studies indicate an in-vivo advantage for the surface modified FD [5, 6, 34]. With the COATING Study, the first multicentric prospective randomized controlled study is enrolled and may clarify the safety and efficacy of coated flow diverters under SAPT [35]. In addition, the ongoing DERIVO 2heal: Clinical Safety and Efficacy of the DERIVO 2heal Embolisation Device study (REheal) should provide further information on the possibility of using SAPT after flow diversion with the D2H. In the future, further manufacturer-independent randomised clinical trials comparing different coatings should be conducted to investigate the safety of SAPT. Despite the possible lower thrombogenicity, the differences of endothelialization and the healing process after flow diversion should be explored. Newly developed optical coherence tomography probes with smaller diameters show promising data. In addition to clearly visualising the apposition of the device, the progress of endothelialisation can also be assessed [36]. This in vivo visualisation should improve our understanding of these processes and perhaps reveal advantages of the surface modifications, such as faster endothelialisation, that we have not yet been able to focus on [37–39].

## Limitations

The retrospective design and data collection without a verification of an independent core lab are the main limitations and could be biased. Also, the heterogeneous data is an issue. The short mean follow-up period (6.6 months) may not be sufficient to fully assess the long-term safety and efficacy of the device, especially considering the potential for late onset complications. Nevertheless, an early high occlusion rate promises even higher occlusion rates in the long term, and the rate of new major device related events after flow diversion is low [40, 41]. On the other hand, to our knowledge, we present the biggest multicentric early experience with the Derivo 2 heal Embolization Device (Acandis, Pforzheim, Germany) in a real-world setting. To verify our presented data, further prospective studies with long term results, such as the ongoing REheal trial (NCT05543447), are necessary.

## Conclusion

The Derivo Embolization Device 2 with HEAL surface modification shows comparable efficacy and safety compared to other coated flow diverting stents with promising short term results. However, to understand the true effect of different coating strategies other study designs are necessary in future trials.

## Supplementary Information

Supplementary Table 1 Patient and aneurysm characteristics for unruptured and ruptured aneurysms, Supplementary Table 2 Treatment parameters, technical difficulties, and occlusion rates for unruptured and ruptured aneurysms, Supplementary Table 3 Antiplatelet medication, Supplementary Figure 1 Comparison of In-stent stenosis in dependency of additional coiling, Supplementary Figure 2 Course of In-stent stenosis
