# Supplementary material for: The DERIVO 2 Heal Embolization Device in the Treatment of Ruptured and Unruptured Intracranial Aneurysms: a Retrospective Multicenter Study
Source: Clin Neuroradiol. 2024 Aug 22;35(1):25–34. doi: 10.1007/s00062-024-01446-8 (PMC11832578; doi:10.1007/s00062-024-01446-8)
Supplement: Supplementary file 1 — Supplementary Table 1 Patient and aneurysm characteristics for unruptured and ruptured aneurysms, Supplementary Table 2 Treatment parameters, technical difficulties, and occlusion rates for unruptured and ruptured aneurysms, Supplementary Table 3 Antiplatelet medication, Supplementary Figure 1 Comparison of In-stent stenosis in dependency of additional coiling, Supplementary Figure 2 Course of In-stent stenosis [file 62_2024_1446_MOESM1_ESM.pdf]

Online supplemental data:

Supplementary Table 1 Patient and aneurysm characteristics for unruptured and ruptured aneurysms

Charachteristics	Unruptured Aneurysms	Ruptured Aneurysms
Age (years) (mean ±*SD* [range])	58.91 ±13.54 (17-90)	57 ±21.99 (25-88)
Sex (n [%])		
Female	57 (75)	5 (62.5)
Male	19 (25)	3 (37.5)
Size (mm) (mean ±*SD* [range])	9.67 ±7.06 (1.8-45)	11.14 ±6.07 (3-21)
Neck width (mm) (mean ±*SD* [range])	5.38 ±3.88 (1.5-25)	6.84 ±5.32 (2-14)
Dome-Neck ratio (mean ±*SD* [range])	1.58 ±0.99 (0.1-8)	1.4 ±0.7 (0.2-2)
Location (n [%])		
Bifurcation	10 (12.3)	0
Sidewall	53 (65.5)	8 (100)
Segmental	18 (22.2)	0
Morphology (n [%])		
Blister like	6 (7.4)	1 (12.5)
Dissecting	3 (3.7)	5 (62.5)
Fusiform	18 (22.2)	0
Saccular	54 (66.7)	2 (25)
Topography (n [%])		
Anterior circulation		
ICA	71 (87.5)	3 (37.5)
MCA	2 (2.4)	1 (12.5)
ACA	1 (1.2)	0
Posterior circulation		
Basilar	5 (6.2)	0
PICA	2 (2.4)	0
SCA	0	1 (12.5)
VA (V4)	0	3 (37.5)

ICA, internal carotid artery; MCA, medial cerebral artery; ACA, anterior cerebral artery; PICA, posterior inferior cerebeallar artery; SCA, superior cerebellar artery; VA, vertebral artery

Supplementary Table 2 Treatment parameters, technical difficulties, and occlusion rates for unruptured and ruptured aneurysms

Characteristics	Unruptured Aneurysms	Ruptured Aneurysms
Additional Coiling (n [%])	22 (28.9)	2 (25)
In-stent balloon angioplasty (n [%])	4 (5.3)	1 (12.5)
Technical difficulties (n [%])	10 (13.1)	
FD did not open	2 (2.6)	0
FD not deliverable	1 (1.3)	0
FD twisted	3 (3.9)	0
Fish mouthing	4 (5.3)	0
Minor adverse events (n [%])	7 (9.1)	
Intraproc. thrombotic event	4 (5.2)	0
Hematoma at puncture side	2 (2.6)	0
CIE	1 (1.3)	

Major adverse events (n [%])	3 (3.9)	1 (12.5)
sICH	2 (2.6)	1 (12.5)
Intraproc. aneurysm rupture	1 (1.3)	0
Major ischemic event	0	0
Time to FU (month) (mean ±*SD* [range])	6.76 (±3.7 [1-18])	5.14 (±1.86 [1-6])
OKM at last FU (n [%])		
A - B	13 (18.3)	2 (28.6)
C - D	58 (81.7)	5 (71.4)

FD, flow diverter; CIE, contrast induced encephalopathy; sICH, symptomatic intracranial hemorrhage, FU, follow-up; OKM, O'Kelly-Marrota Scale

Supplementary Table 3 Antiplatelet medication

Antiplatelet drug	1st Antiplatelet (n [%])	2nd Antiplatelet (n [%])
ASA	79 (94)	3 (3.6)
Clopidogrel	4 (4.8)	57 (67.9)
Prasugrel	0	14 (16.7)
Brilique	0	8 (9.5)
Other	1 (1.2)	1 (1.2)

ASA, acetylsalicylic acid

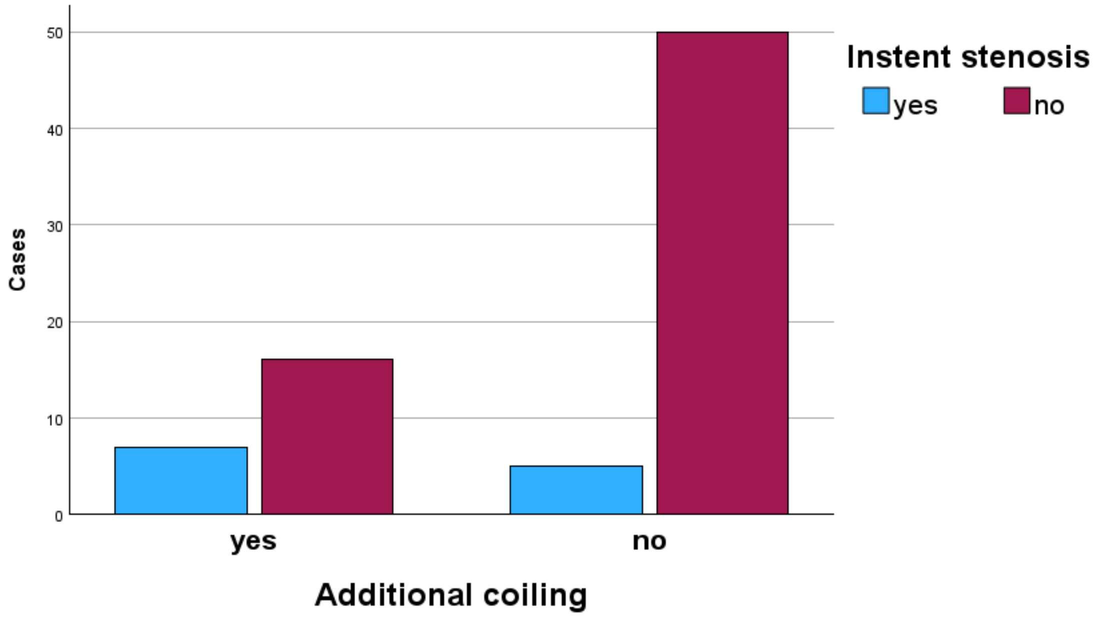

Supplementary Figure 1 Comparison of In-stent stenosis in dependency of additional coiling

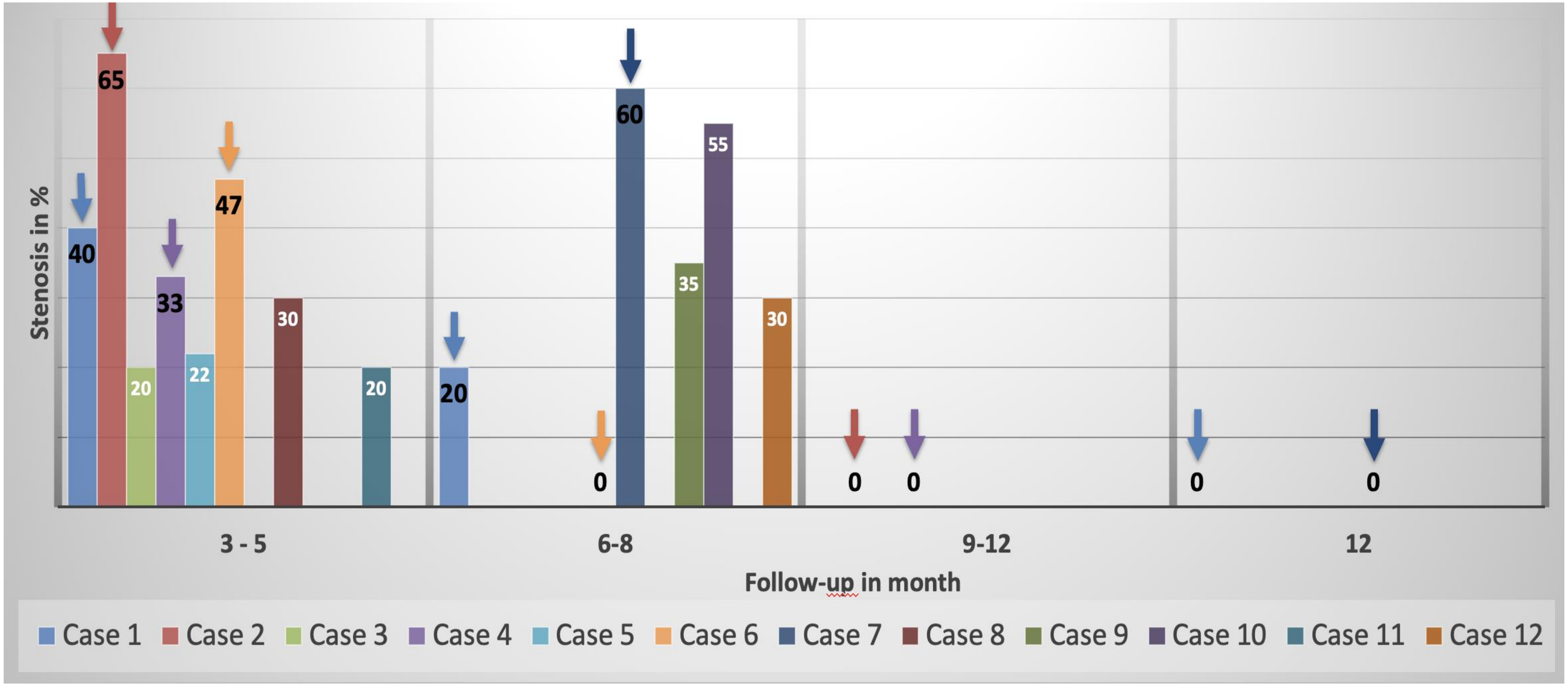

Supplementary Figure 2 Course of In-stent stenosis
